# Impact of Parents’ Knowledge about the Development of Self-Esteem in Adolescents and Their Parenting Practice on the Self-Esteem and Suicidal Behavior of Urban High School Students in Nepal

**DOI:** 10.3390/ijerph17176039

**Published:** 2020-08-19

**Authors:** Ratna Shila Banstola, Tetsuya Ogino, Sachiko Inoue

**Affiliations:** Department of Nursing Science, Graduate School of Health and Welfare Science, Okayama Prefectural University, 111 Kuboki, Soja, Okayama 719-1197, Japan; togino@fhw.oka-pu.ac.jp (T.O.); sinoue@fhw.oka-pu.ac.jp (S.I.)

**Keywords:** adolescent, suicidal behavior, self-esteem, parents, parenting

## Abstract

Background: Suicide is the third leading cause of death in adolescents worldwide, self-esteem is a strong protective factor, and parents may be able to provide interventions. This cross-sectional study aimed to determine how parents can help enhance their adolescent’s self-esteem and prevent suicidal behavior among adolescents in Nepal. Methods: Self-administered questionnaires were distributed to adolescents aged 13–19 years and their parents at eight high schools in three provinces in Nepal (*n* = 575 pairs). The data were analyzed using descriptive and inferential statistics (bivariate and multivariate regression analyses). Results: The mean self-esteem score of adolescents according to the Rosenberg Self-Esteem Scale was 16.59, and the prevalence of suicidal behavior was 11.3%. Parent’s knowledge about the development of self-esteem in adolescents was significantly positively related to parenting practice (communication, support, positive reinforcement, etc.) (B = 1.0, 95% confidence interval, CI 0.89–1.11) and authoritative parenting style (B = 0.2, 95% CI 0.15–0.25). Parental authoritativeness was positively associated with the self-esteem of their adolescents (B = 0.1, 95% CI 0.01–0.18), while adolescents with authoritarian parents were prone to suicidal risk behavior (adjusted odds ratio, AOR = 1.1, 95% CI 1.0–1.19). Conclusion: Counseling to parents based on our findings would be helpful to enhance the self-esteem and prevent suicidal risk behavior in their adolescent children.

## 1. Introduction

Adolescence is a unique developmental period of life marked by the transition from childhood to adulthood in which adolescents have to adjust to various changes and challenges, and this might make adolescents prone to various psychosocial problems [1,2,3,4,5]. Good psychosocial health in adolescence includes having a positive sense of identity and self-worth [6]. In this regard, self-esteem is an important aspect of mental health and it is believed to be one of the predictive factors for the psychological well-being of adolescents in their unique period of identity development [7,8,9,10]. Self-esteem is the overall subjective appraisal of oneself, i.e., positive, negative and mixed thoughts or feelings about one’s own self, and if one has more positive feelings or considers oneself as worthy, self-esteem will be higher [11]. Several studies to date have generated a clearer understanding that adolescents with low self-esteem are at risk for concurrent and future negative outcomes. For example, adolescents with low self-esteem are at risk for depression at present and in their adulthood [12,13,14], delinquency and academic problems [15], risky behavior such as substance use [16,17,18], and risky sexual behaviors [19,20], and long-term unemployment, poor health, economic problems and criminal behavior in adulthood [21]. Studies have also demonstrated that adolescents with low self-esteem are more likely to be at risk of suicide [22,23,24]. Therefore, healthy development with higher self-esteem during childhood and adolescence would lead to healthy and productive adulthood.

Suicide is a serious global problem and not only bears the loss of lives but also substantial economic and social cost. Although it is preventable, suicide is the second leading cause of death among individuals aged 15–29 years old and among girls aged 15–19 years old, and the third leading cause of death among adolescents of both genders worldwide; however, suicide is the leading cause of death among those aged 15–29 years old in both genders in the South-East Asia Region (SEAR) [25]. The majority (90%) of adolescent deaths by suicide have occurred in low-and-middle-income countries (LMICs) [26]. Almost half of adolescent deaths due to self-harm worldwide took place in South Asian LMICs [6]. Nepal is a country in SEAR, with low income, an adult literacy rate of 65.9%, and agriculture as the major occupation. Almost 24% of its total population are adolescents of 10–19 years old. A survey by the World Health Organization (WHO) found the highest rate of suicidal ideation (14%) and high number of suicide attempts (10%) among adolescents in Nepal amongst nations of South East Asia [27]. Although a previous study in Nepal found that self-esteem is a very important protective factor against suicide risk [28], a significant number of Nepalese adolescents were found to have low self-esteem, i.e., 24% to nearly 30% [28,29], and to our knowledge, there has been no study in Nepal that tried to identify how parents can enhance this important protective factor in their adolescent children.

How an adolescent perceives him or herself is presumably based on his/her interaction with others and the interpretation of others, and the starting point of this process is the relationship between the mother/parents and child, followed by peer and other social relationships [1,2,3,30,31]. Parenting does matter, and parents can have an influence on their children, both by genetic makeup and by the way they treat their children [32,33,34]. Bronfenbrenner’s ecological systems theory emphasized the influence of multiple environmental factors on the adolescent’s development, and further placed emphasis on the importance of parents and family through the bioecological model of human development [35]. Therefore, in keeping with these theoretical perspectives, it seems plausible that parents’ knowledge about the development of self-esteem in adolescents, their everyday practice and their parenting style are related to the self-esteem and suicidal behavior of their adolescent children.

The emotional climate in which parents raise their children is known as their parenting style [36]. Baumrind identified three parenting styles, i.e., authoritarian, authoritative, and permissive/indulgent [36,37,38,39,40,41,42]. Authoritative parenting is characterized by warmth, consistency and discipline in mutuality, understanding and support, whereas authoritarian parenting is marked by high control and discipline with punishment, and permissive/indulgent parenting is characterized by very low control and demands, but high degrees of freedom and acceptance [36,39,40,41,42,43]. Past studies revealed the beneficial effect of perceived authoritative parenting on academics [41,44,45,46], the prevention of some risk behaviors such as adolescent smoking and drinking [47,48,49], and increased level of hope in adolescents [50]. However, there have not been many studies on the effect of parenting on the mental health of their children. Although a few studies considered self-esteem, the results of the studies were varied; for example, unlike the findings of studies in the US [51,52], Ghana [48] and Iran [53], a study in Brazil [54] reported that permissive parenting, rather than authoritative parenting, had an optimal effect on the self-esteem of Brazilian adolescents. Furthermore, there was no difference in the effect of these two parenting styles in a study conducted in India [55]. Therefore, the effect of parenting may be dependent on the culture, socioeconomic status and gender, rather than being universal, and may not be able to be generalized to all parts of the world. Furthermore, there have been few studies related to parenting and adolescent suicidal risk behavior. Amongst the few studies, authoritarian parenting was associated with increased suicidal ideation among Jamaican adolescents [56], and father’s authoritarian style was associated with increased suicidal behavior among Lithuanian adolescents [57]; however, authoritarian parenting was not significantly associated with suicidal attempt among German adolescents [34]. Regarding a possible protective effect, a study in the United States indicated that authoritative parenting might prevent suicide attempts because parenting characterized by both high support and boundaries was associated with a reduced number of suicide attempts, and authoritative parenting was found to be more effective in preventing suicide through self-esteem because self-esteem mediated this relationship [58]. However, it is unclear whether these findings from other cultural background are generalizable to the Nepalese context.

Moreover, parenting is a complex concept that includes not only the creation of an emotional climate but also many specific behaviors and characteristics of everyday practice. Although parenting practice and parenting style have been used in studies interchangeably, inclusion of both the parenting practice specific to the outcome of interest and parenting style in studies helps in gaining a better understanding [46,59]. Parents’ practices are a mechanism that directly helps their children attain their specific socialization goal. For example, a parent might be involved with his/her child in assisting with homework, reading with the child at home, etc., so that the child will get good grades in school, while parenting style is the characteristics of parenting or attitude that has an indirect influence on his/her child [36,46]. Therefore, we thought that it was important to include in the present study items such as communication, positive reinforcement, and support, as the overall measure of parenting practice related to the adolescent’s mental development, and more specifically the adolescent’s self-esteem, along with the three parenting typologies (authoritarian, authoritative, and indulgent) to study their associations with the adolescent’s self-esteem and suicidal risk behavior. Thus, the findings will be novel. Similarly, most past studies were based on adolescents’ report of their parents’ practice; however, the parents’ report of their own practice might be different [54,60,61]. Therefore, information generated by parental report of their own parenting will further add to the extant literature. Moreover, a family’s socioeconomic status (SES) is associated with the parents’ goals and values of socialization that they want to teach their children. Parents vary in expectations, and parents may show different behaviors or create a different environment depending on their SES [33,38,62]. This demonstrates the need to include SES while examining the effect of parenting on adolescent outcome. A previous study in Jamaica also recommended this [56]. On the other hand, most studies have been conducted in Western developed nations. Hence, this study in Nepal filled the context, methods and knowledge gaps in existing literature, i.e., the findings from a developing country of SEAR helped to add an understanding on the impact of parenting on adolescents’ outcome of self-esteem and suicidal behavior in a setting/context which is different than that of western nations or the nations of other regions. Furthermore, on the methodological approach, the study included the questionnaire on both the parenting practice and style, and the study has tried to confirm the effect of parenting, that is either the parents’ report will have the same or a different effect on adolescents’ outcome as those reported by previous studies because most of the past studies were based on adolescents’ report. We also believe that the present study has added in the sparsely explored phenomena, i.e., suicidal behavior of adolescents in relation to their parents. Moreover, to add new knowledge, this study has also explored the relationship between parent’s knowledge on an adolescent’s self-esteem and their practice, and to our best knowledge, that has been not yet explored in previous studies. Ultimately, this is the first study in Nepal that has applied the concept of parenting and elucidated the impact of parents’ knowledge and practice on their adolescents’ mental health outcomes, i.e., self-esteem and suicidal behavior. Therefore, the findings will have important implications and contribute to the mental health of Nepalese adolescents through intervention at the parents’ and family level. Our findings will provide the areas that need to be focused on in parental counseling or planning interventions by health workers, school and community health nurses, counselors and others who are involved or interested in the area of adolescent development in Nepal. The study will further be helpful for students and researchers who carry out future studies in the field.

This study was based on three research questions. First, does parents’ knowledge about the development of self-esteem in adolescents have a relationship with their parenting? Secondly, what are the effects of parent’s knowledge about the development of self-esteem in adolescents and parenting practice on the self-esteem of their adolescent children? Third, which parenting type predicts suicidal risk behavior in adolescents?

## 2. Materials and Methods

### 2.1. Study Design, Setting and Population

We conducted a cross-sectional study in Nepal. Administratively, Nepal is divided into seven provinces, and, for this study, three of these provinces were selected, i.e., Kathmandu district, Kaski district, and Palpa district, by the multistage cluster sampling technique. According to the Nepal Ministry of Education (2017) [63], the total number of higher secondary schools in these three provinces was 978, 561, and 532, respectively. We selected three schools (two government and one private) in Kathmandu, three schools (one government and two private) in Kaski, and two schools (one government and one private) in Palpa. Classes nine to eleven of those schools were the final clusters, and adolescents aged 13–19 years and their parents (who were available and willing to participate) were the participants in this study. All students in the selected classes who were present in school on the day of the survey and who agreed to participate in the survey were selected as participants in this study. Adolescents of 13–19 years of age who agreed to participate in the survey were included in the study. Furthermore, we matched the data of the students with the data of their parents; therefore, if both the adolescent and his/her parent completed the respective questionnaires, they were included in the final analysis. This study was approved by the institutional review board of Okayama Prefectural University in Soja, Japan (18-48/2018), and formal permission to conduct the study was obtained from the school authorities at each of the eight higher secondary schools in Nepal.

### 2.2. Data Collection and Analysis Procedure

Data from adolescents was collected in their classrooms of the respective schools. One of the authors visited the students in the classrooms of the schools, explained the study, and obtained assent for participation in the study from the students. Students were asked to fill out the questionnaires and return them to the researcher. The students were asked to give to their parents a well-sealed envelope that contained a detailed explanation of the study, a consent form and questionnaire. The students were asked to bring back to school the questionnaires that their parents had filled out, in the subsequent two or three days. In the letter to the parents, a detailed explanation of the study was provided, and we asked either the adolescent’s mother or father to fill out the questionnaire. Precautions were taken throughout the study at every step to safeguard the rights and welfare of all respondents, and they were assured that their identities will not be disclosed, and the information will be used only for this research purpose. The respondents were given full authority to withdraw their participation from the study without any fear at any time during the investigation. Anonymity was maintained by asking them not to write their names on the questionnaire; however, to match the adolescent’s data with his/her parent’s data, the same code number was used for the adolescent and his/her parent.

Two self-administered questionnaires (one for adolescents and one for their parents) were used in this study. The questionnaire for adolescents consisted of socio-demographic information (age, sex, education level, type of school, school district, etc.) and suicidal risk behavior. Regarding suicidal risk behavior, they were asked about suicidal thoughts, plans, or attempts in the previous 12 months based on a survey questionnaire for adolescents’ risk behavior in South Asia, including Nepal [25], and the response for each item was dichotomized as yes or no, and coded as 1 for those who responded yes and 0 for no. To measure the self-esteem of adolescents, the Rosenberg Self-esteem Scale (RSES) was used. This tool had been reported to have good reliability among Nepalese adolescents in a previous study [29]. The RSES is a 10-item self-report measure consisting of 5 positively and 5 negatively worded items to be answered on a four-point scale ranging from ‘strongly agree’ (score: 3) to ‘strongly disagree’ (score: 0), with the total score ranging from 0 to 30 with reverse scoring for negative items. The higher the score, the higher the self-esteem. We considered an RSES score of <15 as low self-esteem according to the available literatures [29,64,65,66,67].

The questionnaire for parents consisted of three parts: part one included sociodemographic information (age, sex, religion, ethnicity, education level, occupation, economic status, marital status, type of family, etc.). Part two consisted of 12 items that measured their knowledge about the development of self-esteem in adolescents; the score for each item ranged from 1 to 4 and the total score ranged from 12 to 48. Part three included items regarding parenting practice and parenting style. The items on parenting practice included 21 items on a four-point Likert scale; the score for each item ranged from 1 to 4 with a total score ranging from 21 to 84. The items on parenting practice asked about positive reinforcement, open/honest communication, support, etc. (with items such as: usually sit together and maintain open communication with their child; try to be realistic and honest with their child; attend school events and meet schoolteachers; try to make the child feel proud by praising; family has talks and mealtimes together; try to make the child feel that his/her parents are there to help, etc.). The items on parenting style were rated on a five-point Likert scale, with the score on each item ranging from 1 to 5. There were 4 items on authoritarian parenting as strict parents (e.g., whenever my child shows disobedience, I scold and criticize him/her with bursting anger; I have little patience to tolerate any disobedience and have clear expectations); 5 items on authoritative parenting as democratic parents (e.g., I have set some appropriate rules for him/her and give friendly corrections whenever necessary; My child talks with me out of being punished after he/she has done something wrong); and 4 items on Indulgent parenting as laissez-faire parents or freedom-giving parents (e.g., I always threaten my child but do not actually punish him/her; My child is quite free and I do not have any demands or control). The range of total scores on the sections of the four-item authoritarian parenting, five-item authoritative parenting, and four-item indulgent parenting was 4–20, 5–25 and 4–20, respectively. We had developed the questionnaire items regarding the parent’s knowledge about the development of self-esteem in adolescents based on the following literatures: [1,2,3,4,7,11,30,31,68]; we had developed the items on parenting practice related to self-esteem based on the following literatures: [7,11,33,36,56,68,69,70,71,72,73]; and we had developed the items on parenting style based on the following literatures: [39,40,42,43,44,45,47,48,49,50,56,60,74,75,76]. The tool was developed in Nepali. Pretesting was performed on 96 adolescents and 64 parents who had similar characteristics as those of our study participants. Then, modifications were made to make the questions easy to understand and answer. The final data showed good reliability. Cronbach alpha values were 0.89 for the knowledge questionnaire, 0.93 for parenting practice, and in the three parenting-style subscales, 0.63 for authoritarian, 0.78 for authoritative, and 0.68 for indulgent parenting. To ensure the validity of the tools, exploratory factor analysis with maximum likelihood ratio and varimax rotation was executed and found that all 12 items in the knowledge questionnaire had factor loadings of > 0.48 to 0.75 with a single component matrix. Similarly, the 21 items in the parenting practice questionnaire were also extracted in a single component matrix with factor loadings of >0.51 to 0.73. For parenting style, 5 items for authoritative (0.51 to 0.73), 4 items for authoritarian (0.37 to 0.76), and 4 items for indulgent parenting (0.44 to 0.68) were loaded. Upon receipt of the questionnaire from each respondent, the questionnaire was checked for completeness and consistency. Careful attention was paid to the code number to match the questionnaires filled out by the adolescent and by his/her parent. A total of 934 questionnaires were distributed to the parents and 589 were returned (response rate, 63%). However, questionnaires with missing answers were omitted, and 575 pairs (62%) of questionnaires from the adolescents and his/her parents were utilized in the final analysis. Then, statistical analyses were performed with SPSS version 26 (IBM Japan, Tokyo, Japan). Descriptive statistics and inferential statistics (bivariate and multivariate linear/logistic regression analysis) were used. *p* < 0.05 was considered to indicate statistical significance, and 95% confidence intervals were also obtained.

While socio-demographic variables such as, age, sex, SES, ethnicity, religion, parental marital status, type of school and area of residence (school districts) were considered as confounding variables. Previous studies had indicated the associations of these variables with the self-esteem [28,29,52,53,73,77,78] and suicidal behavior of adolescents [24,28,34,56,57], and it was reported that these variables can influence or moderate the relationships between the independent and outcome variables. Using the chi-squared test, we found associations between the adolescent’s gender and the self-esteem level of the adolescents, and between the family’s SES and the adolescent’s self-esteem level; Moreover, the adolescent’s gender and type of school were associated with suicidal behavior. Literatures also indicated that the influence of parenting on adolescent outcome will be different according to the family’s SES and ethnic/cultural background [37,38,59,61,79]. Therefore, we included these basic demographic variables in our regression model to examine the strength of the association between our independent and dependent variables.

### 2.3. Analysis

Before performing regression analysis, we confirmed the statistical assumption of normal distribution of the data with its kurtosis and skewness value [80]. The present investigation was concerned with two outcome variables, i.e., self-esteem and suicidal behavior of adolescents, and their parents’ knowledge about the development of self-esteem in adolescents, parents’ practice and three parenting styles were the independent variables. We examined three hypotheses: (1) parents’ knowledge about the development of self-esteem in adolescents positively predicts their parenting practice and positive parenting style, i.e., authoritative parenting style; (2) parents’ knowledge about the development of self-esteem in adolescents, their parenting practice and authoritative parenting style positively predict the self-esteem of their adolescents; and (3) parents’ practice and parenting style significantly predict suicidal behavior.

In the analysis, at the first step cross tabulation was used to describe sociodemographic factors, and we performed the chi-squared test to study their association with the self-esteem level and suicidal behavior of their adolescent children. Secondly, the relationship between parents’ knowledge about the development of self-esteem, and parenting practice and three parenting styles were assessed with a single linear regression model. Similarly, the relationship between parent’s knowledge about the development of self-esteem, practice and three parenting styles, and self-esteem score of adolescents was analyzed with a binary linear regression model. Thirdly, the relationship among the score of parenting practice and three parenting styles, and the suicidal behavior of adolescents was evaluated using a logistic regression model. Finally, these three crude models were adjusted for possible confounders.

## 3. Results

A total of 575 pairs of adolescents and their parents were participants of the study, considering the completeness of the questionnaires. Table 1 reveals that the participation rate of mothers was higher than that of fathers (61% vs. 39%). The mean age of the adolescents and their parents was 15.82 years and 40.75 years, respectively. The number of girl student was higher than the boys, i.e., 56.2% and 43.8% respectively. Regarding ethnicity, 50.9% belonged to the Janajati ethnic group. Majority were belonged to Hindu religion (82.3%). In relation to the family type, participants from single family were 54.3%, i.e., characterized by the parents and their children are living together, whereas the joint family is marked by the family also include grandparents, uncle, aunt and cousins were 45.7%. About parents’ education level, 52.2% had completed higher secondary and above level, and 47.8% were under the category of literate to secondary level. The majority of the parents—that is, 90.3%—were married and living together, only 9.7% were either widow/widower or divorced/separated. Furthermore, 62.3% reported their family income is sufficient for their livelihood, 12% had hardly sufficient and 25.7% reported they have some surplus/savings.

Male adolescents had a significantly higher self-esteem score than female adolescents. Adolescents whose families had higher SES had a significantly higher self-esteem score. Although statistically not significant, the self-esteem score was higher among adolescents who belonged to the Brahmin/Chhetri ethnicity (71.5%), among adolescents living in Palpa district (76%), among adolescents living with joint family (73.9%), and among adolescents whose parents live together (70.5%). The prevalence of suicidal risk behavior among the adolescents was 11.3% and was significantly higher among female adolescents than among male adolescents, and among adolescents who attended private schools than among those who attended government/public schools.

Adjusted, Adjusted for parent’s age, sex, ethnicity, religion, family type, education, marital status, socioeconomic status, adolescent’s age, and adolescent’s sex

Table 2 shows that significant positive associations were observed between the score on parents’ knowledge about the development of self-esteem in adolescents and the scores on their parenting practice and authoritative parenting. Each increase in knowledge score was related to an increase in practice score (B = 1.0, 95% CI 0.89–1.11), and parents with higher knowledge about the development of self-esteem in adolescents were more likely to adopt the authoritative parenting style (B = 0.2, 95% CI 0.15–0.25).

Table 3 displays that the mean score of self-esteem in the adolescents was 16.59 ± 0.16 (standard error). The mean scores of the following independent variables were: parents’ knowledge 40.47, parenting practice related to adolescent’s self-esteem 70.36, and three types of parenting style, i.e., authoritarian, authoritative, and indulgent, were 13.12, 19.71, and 12.26, respectively. Linear regression analysis revealed a significant bivariate association of the scores on parents’ practice and authoritative parenting style with the adolescents’ self-esteem score, but on multivariate analysis the score on parents’ practice was confounded by two covariates, i.e., socioeconomic status and gender of the adolescent. Although the score on parents’ knowledge about the development of self-esteem in adolescents was associated with the score on parenting practice, it did not show any association with the self-esteem score of the adolescents. However, the score on authoritative parenting showed a beneficial effect on the adolescent’s self-esteem score after adjustment for several covariates related to the parents and adolescents (B = 0.1, 95% CI 0.11–0.18).

Table 4 shows the results of multivariate logistic regression analysis, that is parental authoritarian style was a significant risk factor for suicidal behavior in the adolescents (AOR = 1.1, 95% CI 1.0–1.19). Although the scores on authoritative and indulgent parenting styles showed inverse relationships with suicidal risk behavior, they were not significant statistically.

Table 5 reveals the result of univariate analyses, significant relationships were found between economic status, occupation of mother, and area of residence/school district, and authoritative parenting. Homemaker mothers were more likely to be authoritative with their child, whereas, if family income was hardly sufficient for livelihood, the parents in such homes were less likely to be authoritative. Regarding the school district, Palpa district is a small district with a small urban area in the hill region of Nepal that differs in terms of family structure, neighborhood and parents’ occupation status from the other two districts. In Palpa district, most of the mothers stay at home and are homemakers, and most families have their own agricultural land and grow their own crops; therefore, livelihood is easier in Palpa district compared with the livelihood of working parents who stay in rental properties in Kathmandu and Pokhara/Kaski. The parents in Palpa district were more authoritative than the parents in Kathmandu and Kaski.

## 4. Discussion

In this study, we examined the effect of parenting practice according to the parents’ report on the self-esteem and suicidal behavior of adolescents according to the adolescents’ report. Furthermore, we used the SES as reported by the parents (which is considered to be a more appropriate and reliable approach than adolescents’ report of their family’s SES) as a covariate in the analysis of the relationship between parenting and adolescent outcome (i.e., their self-esteem and suicidal behavior). Nevertheless, given the strong tradition in Nepalese culture that favors boys over girls, and based on the reported evidence of higher rates of suicidal behavior and low self-esteem among adolescent girls in Nepal, it was also worthwhile to consider gender as another important covariate. Here, we would like to discuss our findings based on the outlined research questions, with important practical implications at the levels of the family/parents, school/public health nurses, psychologists, counselors and others who work in the arena in contributing to the mental health of adolescents. Most importantly, programs can be developed to provide effective counseling for the parents of adolescents, parenting training, and knowledge so that parents can enhance their adolescent’s self-esteem and prevent suicides.

In our study, the mean self-esteem score of adolescents was 16.59, which is similar to those obtained in previous studies in Nepal [29,81]. Further in line with the previous studies, we found that girls were more likely to have lower self-esteem than boys [28,29,52,79]. The prevalence of suicidal behavior found in this study (11.3%) was similar to those found in previous studies [27,82].

Regarding our first research question as to whether parents’ knowledge about the development of self-esteem in adolescents has a relationship with the parenting practice of parents, linear regression analysis proved our assumption that revealed that the parenting practice score was significantly associated with the knowledge score; that is, parents with higher knowledge about the development of self-esteem in adolescents had a better practice score (on overall communication, relation, involvement, support, and positive reinforcement, i.e., appreciation, praise). Similarly, parents with higher knowledge about the development of self-esteem in adolescents were significantly more likely to practice an authoritative parenting style. In this regard, our findings on the relationship between knowledge and practice suggested that if parents have knowledge about the development of self-esteem in adolescents, they practice good communication and have a good relationship with their children, try to be supportive, practice positive reinforcement in the form of praise, appreciation and encouragement for good effort, and try to be more involved or participate in academic and other areas of their child’s life. Parents with better knowledge about the developmental aspect of adolescents are likely to be more authoritative, i.e., have open bidirectional communication and respect the views of their child, involve the adolescent in decision making, set rules and make corrections in a mutual way, being less focused on punishment, and manage time to be engaged with his/her child and his/her academic and school activities, etc., as much as possible. Previous studies in different parts of the world showed that authoritative parents are more likely to encourage their children and engage in their school activities [46]. Parental involvement [8,16], parental acceptance and support [24,64,83], and having a good relationship with their children [37,70,72] are beneficial for their children’s self-esteem.

Our linear regression analysis also provided the answer to our second question, revealing that the authoritative style of parenting according to the parent’s report was positively associated with their adolescent’s self-esteem. Several past studies reported that adolescents who perceived that their parents are authoritative were significantly more likely to have higher self-esteem [50,51,52,53]. With this finding, we could add to the extant literature that the parental report of their parenting style also has the same beneficial effect as the parenting style perceived by adolescents in previous studies. Parents can contribute to the development of self-esteem in their adolescent children through authoritative parenting in Nepal as well. While another study showed that permissive parenting contributed more to the self-esteem of adolescents compared with authoritative parenting [54], in the present study, permissive parenting did not show a beneficial effect on the self-esteem of Nepalese adolescents. Hence, this discrepancy may have been due to differences in the setting and context [61]. Furthermore, although authoritative parenting had a beneficial effect as seen in most previous studies, it should be noted that the effect of SES followed by gender was also strong in the Nepalese context. In addition, while we explored factors associated with authoritative parenting, we observed significant associations between authoritative parenting and economic status, occupation of the mother, and area of residence. A past study in Turkey also provided partial support for the association between SES and perceived parenting style [62]. Parental goals and values of socialization of their children, and their expectations from the child may be different in different socio-economic strata, and parents of different SES may show different behaviors or create a different environment for their children. Parents of higher SES are more likely to be democratic and assertive [38,61]. Similarly, regarding mother’s occupation, homemaker mothers may have more time to be involved and interact with her child. Compared to working mothers, homemaker mothers may have less job-related/dual career stress, and as such they may have a more positive approach with their child. According to this notion, to support this finding, past evidences have suggested that the parenting style in higher SES homes was found to be more democratic and accepting, and these parents value self-direction in their children and exhibit more warmth and involvement, while in families with lower SES, working-class parents are more likely to be harsh, punitive, and oriented towards order and obedience, i.e., the authoritarian style [38,46]. Regarding the school district, parents in Palpa district were more authoritative compared to parents in Kathmandu and Kaski. Palpa is a hill/countryside small urban area of Nepal, whereas Kathmandu is the capital city and Kaski is the second largest city in Nepal after Kathmandu, and more parents are employed outside their home. On the other hand, compared to large cities, parents in rural or small urban areas might have less expectations for their child about their future. This might be the reason that they are less likely to be harsh and punitive, and are more accepting. A future study should explore the exact cause of this difference.

We also found that the parenting practice score of parents (i.e., the total score derived from items such as communication, relationship, support, participation, appreciation, encouragement, etc.) was positively associated with the self-esteem score of their adolescents on bivariate analysis, but this was completely confounded by two variables, i.e., SES and gender, on multivariate analysis. Socioeconomic status had the stronger effect on this relationship. Therefore, it can be added that the SES and the environmental context might influence the goals, practice and style of parents because children do not grow and develop and the adult does not parent in isolation, but within the surrounding culture, economy and context [38], and the effect of parenting is not universal but is dependent on culture and gender [37]. It was also reported that adolescents perceive a difference in parenting by gender and SES [62]. Therefore, researchers should consider different cultures and contexts when studying the relationship between parenting and self-esteem of their children [79]. Past studies showed that adolescents who perceived that they had higher family SES had higher self-esteem [73,78], and our study also revealed that socioeconomic status mattered. On the other hand, a strong male-dominant social culture and religion still play a role in the difference in the rearing pattern of male and female children and the view of society in Nepal. Nepalese adolescents have high rates of health and social vulnerabilities and psychosocial problems, and they are especially higher among girls. Furthermore, the psychosocial well-being of adolescents is also affected by their SES in Nepal [84]. Despite efforts to reduce gender inequality, women/girls in Nepal are still marginalized in society (e.g., discrepancy in the available roles, priorities, opportunities, and resources) which affects their health, development and well-being [85]. Another study on resilience in Nepalese adolescents indicated that one-fifth of the students had low resilience and girls had a lower total resilience score [86]. Here, in this single cross-sectional study, with our main aim of how to promote self-esteem and prevent suicidal behavior in adolescents, we could not determine the exact rationale for the effect of SES on parents’ practice; thus, a future study should explore why this is the case. Currently, based on our findings, it can be said that although gender is not modifiable and SES is a complex factor, Nepalese parents can enhance their adolescent’s self-esteem through authoritative parenting practice.

Thirdly, regarding the suicidal risk behavior of adolescents, our multivariate logistic regression analysis revealed that the parental authoritarian style was a significant risk factor for suicidal behavior in adolescents. Although none of the other variables (parents’ practice, and authoritative and indulgent parenting styles) was a significant factor, the odds of those factors were in line with their protective effect. To our knowledge, there are a limited number of articles on the impact of parents on their adolescent’s suicidal behavior; however, to support this findings a study in Jamaicans indicated that adolescents who reported that their parents are more authoritarian were more prone to suicide ideation and gender had a moderating effect [56]. Among Lithuanian adolescents, some manifestations of suicidal behavior were significantly associated with low satisfaction with family relationships, low emotional support from their mothers and fathers, low monitoring by their mothers, low school-related parental support, and authoritarian-repressive parenting style of their fathers [57]. However, the results of the present study are in contrast with the results of the study from Germany [34] that reported that authoritarian parenting was not a significant predictor of suicide attempt among German adolescents; however, it should be noted that only ninth-grade adolescents were included in their study, and the difference in country context might have caused this discrepancy. The effect of parenting style or the perception of their parents’ parenting style by adolescents could differ between developed and undeveloped nations [34,59]. Moreover, a study in Czech adolescents of 11–16 years of age indicated that weak control and more warm relations can have a reducing effect on adolescent’s self-harm behavior [87]; these are characteristics similar to the authoritative style of parenting. Similarly, another study in the US reported that both higher support and boundaries by parents for their children, which are also characteristics of authoritative parenting, were found to be protective against suicidal behavior among adolescent students of 7th and 9th grades, and on top of this relationship, the self-esteem of the adolescents had a mediating effect [58]. Therefore, parents can lower suicidal risk in their adolescent children by enhancing their self-esteem through authoritative parenting while providing higher support and maintaining a good relationship with their adolescent children. However, our analysis did not prove the significant association of parental authoritativeness with reduction in suicidal risk behavior, although the odds were in line with a protective effect. Similar to previous studies, we established in the Nepalese context that authoritarian parenting is a risk factor for suicidal behavior. In support of our findings, studies on parenting and adolescent outcome have stated that parenting with high control and harsh parenting, i.e., the authoritarian parenting style, lead to negative outcomes in their adolescent children, such as depression, diminished academic success, and self-harm, while authoritative parenting has a beneficial effect [41,43,44,45,46,47,48,49,50,51,52,61,83,88]. Hence, our findings suggested that parents can prevent suicidal behavior in their adolescent children by reducing their authoritarian parenting style. Parents should focus on reducing negative criticism and punishment-oriented behavior towards their adolescent children; rather, they should focus on correction of any misbehavior by positive mutual communication and understanding, and by creating a less threatening environment. It seems that parents need to control their burst of anger and need to listen/analyze or better try to understand the rationale from the perspective of the child/adolescent if any problem, disobedience or breaking of set rules occurs. However, a future study to establish a cause–effect association and longitudinal studies to further support these findings are needed. The Freudian view is that adolescence is a period of emotional upheavals, Erikson’s view is that adolescence is a period of identity crisis, and Jessor’s view is that adolescence is a period of problem behavior associated with personal and multiple external factors. Although teenagers spend much more time with their peers than with their parents and may sometimes openly challenge their parents’ actions and beliefs, still they value their relationships with their parents tremendously and adolescents are not always the critical ones, but rather the ones to be molded [1,2,3,4,30,31,32,33,68]. Therefore, parents should be counseled to help them understand the undeniable important fact that they are the best and most important resources for adolescent’s positive development.

### Strengths and Limitations of This Study

The findings of this study should be interpreted in light of its limitations. Due to the cross-sectional design of this study, causality cannot be determined. This study was conducted in urban high schools; therefore, the findings would not apply to the understanding of adolescents and parents living in rural areas or adolescents who do not attend formal schools. The information was collected by self-report measure; therefore, our findings did not help in our understanding of illiterate parents, our understanding of those parents who did not respond to the questionnaire, and our understanding of those adolescents who were absent on the day of data collection or who did not completely fill out the questionnaire. Only either the adolescent’s father or mother participated in the study, and there was a possible influence from the parent who did not participate in this study; therefore, a future study should consider collecting data from both parents to gain a wider understanding. Furthermore, we included only those participants in which both the adolescent and his/her parent filled out the questionnaires completely because we needed to match the data of the adolescent with the data of his/her parent. Despite these limitations, the first study being in Nepal, this study would make contributions to practical implications as well as the literature in the country context. This study yielded important information covering a large geographic area, i.e., three provinces of Nepal. We also hope that this study adds to the extant literature on parenting and adolescent mental health. The information on parenting practice was provided by parental report rather than by mere perception of adolescents, and we tried to fill the gap by taking into account the effect of SES according to the parent’s report on their parenting practice and adolescent’s outcome. Moreover, this study revealed the picture of parental factors that affect adolescents’ mental outcomes (i.e., self-esteem and suicidal behavior) from the context of diverse social, religious, cultural, ethnic and economic backgrounds.

## 5. Conclusions

A significant number of adolescents in Nepal have low self-esteem and show suicidal risk behavior, although parents’ knowledge about the development of self-esteem in adolescents was not significantly associated with the self-esteem level of the adolescents; however, parents with greater knowledge about the development of self-esteem in adolescents were significantly more likely to have better parenting practice, and more likely to adopt authoritative parenting. Importantly, authoritative parenting positively predicted their adolescent’s self-esteem; on the other hand, authoritarian parenting was a risk factor for suicidal behavior. Study covariates such as SES and gender showed strong effects. Gender is not modifiable and SES is complex, but parents can contribute to the self-esteem of their adolescents by adopting an authoritative style, and by reducing their authoritarian parenting, parents can contribute to prevention of suicidal behavior. Hence, we conclude that parents can play a very effective role in promoting positive mental health in their adolescent children. School health nurses, community/public health nurses, psychologists, counselors and others who work in the area of adolescent mental health and development can focus on planning knowledge intervention, parenting training or counseling for the parents of adolescents.

## Figures and Tables

**Table 1:** Characteristics of the parents and adolescents according to the self-esteem level and suicidal risk behavior of the adolescents (*n* = 575).

Variable	Total		Self-Esteem Level *		*p*-Value ^#^	Suicidal Risk Behavior **		*p*-Value ^#^
	No.	%	Low (*n* = 172)	Normal-High (*n* = 403)		No (*n* = 510)	Yes (*n* = 65)	
**Parents’ characteristics**								
**Age**								
≤40 years old	338	58.8	102 (30.2)	236 (69.8)	n.s.	299 (88.5)	39 (11.5)	n.s.
≥41 years old	237	41.2	70 (29.5)	167 (70.5)		211 (89.0)	26 (11.0)	
Mean ± SD (40.77 ± 6.31)								
**Relation with child**								
Father	226	39.3	67 (29.6)	159 (70.4)	n.s.	200 (88.5)	26 (11.5)	n.s.
Mother	349	60.7	105 (30.1)	244 (69.9)		310 (88.8)	39 (11.2)	
**Ethnicity**								
Brahmin/Chhetri	215	37.4	60 (27.9)	155 (72.1)	n.s.	189 (87.9)	26 (12.1)	n.s.
Janajati	293	50.9	91 (31.1)	202 (68.9)		260 (88.7)	33 (11.3)	
Others	67	11.7	21 (31.3)	46 (68.7)		61 (91.0)	6 (9.0)	
**Religion**								
Others	102	17.7	30 (29.4)	72 (70.6)	n.s.	89 (87.3)	13 (12.7)	n.s.
Hindu	473	82.3	142 (30.0)	331 (70.0)		421 (89.0)	52 (11.0)	
**Province/District**								
Province 3 Kathmandu	261	45.4	88 (33.7)	173 (66.3)	n.s.	234 (89.7)	27 (10.3)	n.s.
Province 4 Kaski	217	37.7	60 (27.6)	157 (72.4)		190 (87.6)	27 (12.4)	
Province 5 Palpa	97	16.9	24 (24.7)	73 (75.3)		86 (88.7)	11 (11.3)	
**Type of family**								
Single	312	54.3	103 (33.0)	209 (67.0)	n.s.	274 (87.8)	38 (12.2)	n.s.
Joint	263	45.7	69 (26.2)	194 (73.8)		236 (89.7)	27 (10.3)	
**Education level (*n* = 548)**								
Literate to secondary level	262	47.8	83 (31.7)	179 (68.3)	n.s.	235 (89.7)	27 (10.3)	n.s.
Higher secondary and above	286	52.2	81 (28.3)	205 (71.7)		249 (87.1)	37 (12.9)	
**Marital status**								
Separated/Widow/Widower	56	9.7	19 (33.9)	37 (66.1)	n.s.	53 (94.6)	3 (5.4)	n.s.
Together	519	90.3	153 (29.5)	366 (70.5)		457 (88.1)	62 (11.9)	
**Socio-economic status**								
Hardly Sufficient	69	12.0	26 (37.7)	43 (62.3)	0.000	62 (89.9)	7 (10.1)	n.s.
Sufficient	358	62.3	121 (33.8)	237 (66.2)		316 (88.3)	42 (11.7)	
Surplus	148	25.7	25 (16.9)	123 (83.1)		132 (89.2)	16 (10.8)	
**Adolescents’ characteristics**								
**Age**								
13-15 years	268	46.6	78 (29.1)	190 (70.9)	n.s.	238 (88.8)	30 (11.2)	n.s.
16-19 years	307	53.4	94 (30.6)	213 (69.4)		272 (88.6)	35 (11.4)	
Mean ± SD (15.69 ± 1.31 years)								
**Sex**								
Male	252	43.8	62 (24.6)	190 (75.4)	0.014	234 (92.9)	18 (7.1)	0.005
Female	323	56.2	110 (34.1)	213 (65.9)		276 (85.4)	47 (14.6)	
**Type of school**								
Government/Public	365	63.5	119 (32.6)	246 (67.4)	n.s.	332 (91.0)	33 (9.0)	0.024
Private	210	36.5	53 (25.2)	157 (74.8)		178 (84.8)	32 (15.2)	

Total No., Total number. Number in parentheses indicates percentage. ^#^ Chi-square test. Statistical significance at *p* < 0.05, n.s., not significant. * Self-esteem level of the adolescents. Low self-esteem = score < 15 on the Rosenberg Self-esteem Scale (RSES). ** Suicidal risk behavior of the adolescents.

**Table 2:** Linear regression association between parents’ knowledge about the development of self-esteem in adolescents and their parenting practice.

	Parenting Practice (Communication, Support, Reinforcement, etc.)				Authoritarian Parenting				Authoritative Parenting				Indulgent Parenting			
	Unadjusted		Adjusted		Unadjusted		Adjusted		Unadjusted		Adjusted		Unadjusted		Adjusted	
	B	β	B	β	B	β	B	β	B	β	B	β	B	β	B	β
	95% CI		95% CI		95% CI		95% CI		95% CI		95% CI		95% CI		95% CI	
Parent’s knowledge about the development of self-esteem in adolescents	1.0	0.56	1.0	0.59	0.01	0.02	0.02	0.03	0.18	0.27	0.20	0.30	0.03	0.04	0.05	0.07
	(0.85, 1.08) **		(0.89, 1.11) **		(−0.03, 0.06)		(−0.03, 0.07)		(0.13, 0.23) **		(0.15, 0.25) **		(−0.03, 0.08)		(−0.01, 0.10)	

** significant *p* = 0.000; B, unstandardized coefficient; β, standardized coefficients beta; CI, confidence interval.

**Table 3:** Linear regression association of parents’ knowledge about the development of self-esteem in adolescents and parenting practice with the self-esteem level of the adolescents.

Independent Variables				Self-Esteem Level of Adolescents									
			Unadjusted						Adjusted				
	Mean (SE)	B	β	*t*	*P*	LCI	UCI	B	β	*t*	*P*	LCI	UCI
Knowledge about the development of self-esteem in adolescents	40.47 (0.23)	0.051	0.073	1.745	0.082	−0.006	0.108	0.053	0.075	1.784	0.075	−0.005	0.111
Parenting practice (communication, support, reinforcement, etc.)	70.36 (0.39)	0.036	0.087	2.101	0.036	0.002	0.069	0.024	0.058	1.361	0.174	−0.011	0.059
Authoritarian parenting	13.12 (0.13)	−0.050	−0.043	−1.019	0.309	−0.146	0.046	−0.040	−0.034	−0.799	0.424	−0.139	0.059
Authoritative parenting	19.71 (0.15)	0.125	0.121	2.923	**0.004**	0.041	0.208	0.098	0.093	2.213	**0.027**	0.011	0.186
Indulgent/Laissez-faire parenting	12.26 (0.15)	−0.024	−0.023	−0.542	0.588	−0.112	0.063	−0.060	−0.055	−1.309	0.191	−0.150	0.030

SE, standard error; B, unstandardized coefficient; β, standardized coefficients beta; LCI, lower limit of confidence interval; UCI, upper limit of confidence interval. Adjusted, adjusted for parent’s age, sex, ethnicity, religion, family type, education, marital status, socioeconomic status, adolescent’s age, and adolescent’s sex. Adolescents’ self-esteem score: 16.59 ± 0.16 (mean ± SE).

**Table 4:** Logistic regression odds ratios and 95% confidence intervals for factors predicting suicidal behavior in adolescents.

Independent Variables			Suicidal Risk Behavior of Adolescents							
		Unadjusted					Adjusted			
	B	*p*	OR	LCI	UCI	B	*p*	OR	LCI	UCI
Parenting practice (communication, support, reinforcement, etc.)	−1.499	0.552	0.99	0.966	1.019	−3.075	0.628	0.99	0.964	1.022
Authoritarian parenting	−2.922	0.113	1.1	0.985	1.155	−4.621	0.041	1.1	1.004	1.194
Authoritative parenting	−0.910	0.069	0.94	0.884	1.005	−2.482	0.136	0.95	0.885	1.017
Indulgent/Laissez-faire parenting	−1.674	0.377	0.97	0.903	1.040	−3.569	0.897	0.99	0.922	1.074

OR, odds ratio; B, unstandardized coefficient constant; LCI, lower limit of confidence interval; UCI, upper limit of confidence interval. Adjusted, Adjusted for parent’s age, sex, ethnicity, religion, family type, education, marital status, socioeconomic status, adolescent’s age, adolescent’s sex, school district, and type of school.

**Table 5:** Linear regression coefficients and level of significance of the factors associated with authoritative parenting.

Parental Background Variables	Authoritative Parenting Style					
	B	β	*t*	*p*	LCI	UCI
Parent’s age	−0.040	−0.066	−1.592	n.s.	−0.089	0.009
Relation with child						
Father ref.						
Mother	−0.175	−0.023	−0.541	n.s.	−0.812	0.461
Religion						
Others ref.						
Hindu	0.430	0.043	1.038	n.s.	−0.383	1.244
Ethnicity						
Brahmin/Chhetri	0.267	0.034	0.816	n.s.	−0.375	0.910
Janajati	−0.174	−0.023	−0.548	n.s.	−0.796	0.448
Others	−0.186	−0.016	−0.377	n.s.	−1.155	0.783
Family type						
Single ref.						
Joint	0.445	0.059	1.403	n.s.	−0.178	1.069
Educational level						
Secondary and below ref.						
Higher secondary and above	−0.143	−0.019	−0.450	n.s.	−0.767	0.481
Mother’s occupation *						
Home-maker	0.635	0.083	2.003	0.046	0.012	1.258
Father’s occupation						
Works at home/unemployed ref.						
Employed	0.214	0.014	0.330	n.s.	−1.057	1.484
Economic status *						
Hardly sufficient	−1.242	−0.107	−2.564	0.011	−2.194	−0.291
Sufficient	0.211	0.027	0.646	n.s.	−0.431	0.852
Surplus	0.427	0.049	1.181	n.s.	−0.283	1.138
Marital status						
Widow/widower/separated/divorced ref.						
Married and living together	0.161	0.013	0.301	n.s.	−0.888	1.210
Age of adolescent child	0.003	0.001	0.021	n.s.	−0.234	0.239
Sex of child						
Male ref.						
Female	−0.151	−0.020	−0.475	n.s.	−0.778	0.475
School district *						
Kathmandu	−0.618	−0.081	−1.948	n.s.	−1.240	0.005
Kaski	−0.107	−0.014	−0.328	n.s.	−0.749	0.534
Palpa	1.271	0.126	3.030	0.003	0.447	2.095
School type						
Government/Public ref.						
Private	−0.626	−0.080	−1.910	n.s.	−1.270	0.018

B, unstandardized coefficient; β, standardized coefficients beta; LCI, lower limit of confidence interval; UCI, upper limit of confidence interval, * significant factor.
